# Fourier Transform Infrared Spectroscopy to Assess the Degree of Alteration of Artificially Aged and Environmentally Weathered Microplastics

**DOI:** 10.3390/polym15040911

**Published:** 2023-02-11

**Authors:** Claudia Campanale, Ilaria Savino, Carmine Massarelli, Vito Felice Uricchio

**Affiliations:** Italian National Council of Research—Water Research Institute, CNR-IRSA, 70132 Bari, Italy

**Keywords:** microplastics, ATR-FTIR, weathering, freshwater, pristine, degradation indexes, Ofanto river

## Abstract

Fourier transform infrared (FTIR) is a spectroscopy technique widely used to identify organic materials. It has recently gained popularity in microplastic (MP) pollution research to determine the chemical composition of unknown plastic fragments. However, it could also be used to evaluate the degree of ageing of MPs collected from the environment. In this context, the principal aim of our research has been to qualitatively evaluate the natural weathering of environmental MPs collected in an Italian freshwater body (the Ofanto River) using ATR-FTIR technology. Furthermore, we compared environmental particles to weathered artificial MPs under controlled light and temperature conditions and to unaltered pristine materials to assess the results. FTIR spectra were acquired using a Nicolet Summit FTIR (ThermoFisher Scientific) equipped with an Everest ATR with a diamond Crystal plate and a DTGS KBr detector (wavenumber range 4000–500 cm^−1^, 32 scans per spectrum, spectral resolution of 4 cm^−1^). The degree of ageing was assessed using three different indexes known to be related to changes in MPs: Carbonyl Index (CI), Hydroxyl Index (HI), and Carbon-Oxygen Index (COI). The overall results showed that the regions reflecting changes (hydroxyl groups, peaks from 3100 to 3700 cm^−1^, alkenes or carbon double bonds, 1600 and 1680 cm^−1^, and carbonyl groups, 1690 and 1810 cm^−1^) appeared significantly modified in artificial and natural weathered particles compared to the pristine materials. The indexes calculated for polymers degraded under the artificial photo and thermo ageing conditions displayed a general tendency to increase with the time in contact with irradiation time. Particular enhancements of CI of PS fragment and PE pellet, HI of PE and PS fragments and PE pellet, and COI of PS fragment were observed. Otherwise, the following incubation of the same particles at a constant temperature of 45 °C did not further affect the chemical composition of the particles. Moreover, new unique peaks were also observed in the freshwater particles, almost all in the fingerprint region (1500–500 cm^−1^). Differences in CI, HI, and COI were evidenced among the different morphological MP shapes. On the one hand, the CI calculated for the environmental PE pellets showed values ranging from 0.05 to 0.26 with a mean value of 0.17 ± 0.10. Most samples (57%) presented a CI with values between 0.16 and 0.30. On the other hand, fragments presented slicer modifications in the carbonyl region with CI values lower than pellets (0.05 ± 0.05). This index helps evaluate the degradation of PE MPs by UV light, increasing with enhancing residence time in the environment. Conversely, fragments showed greater values of HI (5.90 ± 2.57) and COI (1.04 ± 0.48) than pellets, as well as lines, which presented the maximum value of HI (11.51). HI is attributed to the bond vibrations of hydroxyl, carboxyl, or phenol groups. In contrast, COI is frequently attributed to the vibrations of C_O bonds found in carbohydrates, alkanes, secondary alcohols, and ketones. In conclusion, our results showed characteristics spectra acquired from environmental particles compared to pristine and artificial aged ones. The interpretation of our main results emphasizes the need to conduct ecotoxicological experimental studies using naturally weathered particles due to the unicity of their properties, which are more helpful for understanding microplastic pollution effects.

## 1. Introduction

Fourier transform infrared (FTIR) is a spectroscopy analytical technique widely used to identify organic materials [1]. It has recently gained popularity in microplastic (MP) pollution research to determine the chemical composition of unknown plastic fragments with high reliability, comparing the IR spectrum of an unknown plastic sample with spectra of known polymers [2].

The chemical structures of unknown polymers can mainly be recognized through their specific IR absorption frequency. However, their exact chemical structures can only be deduced from IR spectra if compared with known data or their monomers’ IR spectra.

One of the most critical issues of plastic and microplastic research is their degradation potential, one of the most important factors that can be used to calculate their persistence in the environment in certain conditions [3]. Although plastic has a high resistance to degradation due to the presence of plasticizers, antioxidants, and stabilizers added during the production process, its ageing processes are induced by a combination of the interaction of biotic and abiotic factors. For example, biodegradation (by microorganisms), photodegradation, mechanical stress, and chemical and physical weathering (by air, wave action, wind, temperature, and sand-blasting) favour the creation of cracks, fractures, and alterations of the chemical structure of the plastic surface which produce, in time, a wide variety of particles, called microplastics, differing in shapes and sizes [4,5,6].

The photodegradation of plastic by UV radiation is considered the most important weathering process for the MPs generation, which induces the splitting of chemical bonds, branching, and formation of oxygen-containing groups such as carboxylic, aldehyde, ketone, and hydroxyl [7,8]. The formation of these groups causes an increase in hydrophobicity, polarity, charge, and specific surface area of MPs, also favouring the interaction between MPs and pollutants [9,10,11,12,13].

Weathering has been reported to significantly change the physicochemical properties of MPs, critically affecting the sorption behaviour of pollutants (e.g., hydrophobic organic compounds, antibiotics, and metal ions) onto MPs [7]. These contaminants have direct and documented effects on human health linked to a series of diseases, including cancer and respiratory pathologies [14].

Moreover, ageing processes potentially affect the MPs ingestion by organisms, including humans, due to the increasing size reduction in weathered particles, resulting in more easily ingested and more complex egested particles by biota. Indirect risk to humans and biota can be induced concerning the leaching of toxic additives, which has also been observed in previous studies [14,15]. Great concern regarding the exposition and effects of nano and microplastics on human health is still a controversial debate, including several variables to be considered [16].

In this regard, FTIR is a valuable tool for measuring the changes in chemical bonds typical of MPs ageing (carbonyl groups, hydroxyl, and carbon-oxygen) [7,17]. The information obtained from an IR spectrum is used to support the measurement of several weathering indexes representative of the degree of degradation of polymers [2]. For example, the Carbonyl index has been used as the essential indicator of carbonyl group formation during PP and PE photo or thermo-oxidation in the range of 1850–1650 cm^−1^ [18]. Carbonyl groups (C=O) are, in fact, the main photo-absorbing species responsible for reactions triggered by UV exposure [19].

Previous research [20,21] simulated UV ageing of low-density polyethylene by observing a 30% increase in carbonyl indices. 

Even polypropylene showed an increase in the absorption of hydroxyl and carbonylic groups under the influence of both artificial light (UV-B) and natural sunlight for six months.

Another study [22] simulated a marine water environment to expose polyethylene pellets in the dark and observe morphological and structural changes in the polymer.

However, the complexity of the natural processes requires the simulataneous study of numerous variables, and the exposition times set up in laboratory studies are logistically lower compared to what really occurs in the environment.

If simulations of MPs degradation in the laboratory provide a reasonable basis for understanding the process, the results obtained are often only partially comparable to natural environmental systems.

Most plastic degradation studies were conducted under experimental laboratory conditions to simulate various environmental factors [23,24,25,26,27]. Moreover, they focused mainly on the degradation of plastic in marine environments, while much less attention was given to freshwater, despite the widespread plastic pollution [23,28,29,30]. The freshwater environment is distinguished from the marine environment by the solar light’s intensity, the water’s physicochemical properties, and the biological characteristics [8]. Therefore, it is essential to provide more data on the phenomenon to better understand the fate of MPs in this environment.

To fill in gaps of information between artificial and actual weathering processes, our study’s objective has been to investigate the influence of UV radiation and heating on microplastic weathering, evaluating differences among polymers and morphologies. In addition, the natural ageing of environmental MPs collected in an Italian freshwater body (the Ofanto river) was assessed compared to pristine, artificial, and natural weathered particles. The approach adopted provided the use of ATR-FTIR technology through the calculation of weathering indexes used as a standard approach for estimating the ageing stage of environmental particles. To the best of our knowledge, no studies assessed the ageing of naturally weathered particles collected from a freshwater environment.

## 2. M&Ms

### 2.1. Microplastics Recruitment

#### 2.1.1. Pristine Microplastics Selection

Polymers composed of polyethylene, polypropylene, and polystyrene were selected as pristine materials due to their overall production and dispersion into the environment [31,32].

A red polypropylene plastic cup, a black polyethylene mulch sheet, and a white polystyrene box were cut in the laboratory to obtain fragments of virgin MPs (Table 1). Particles were sieved to select a size between five and one mm. Furthermore, resin pellets of five mm composed of PE and PP were purchased from the local plastic industry and added to the other homemade pristine particles for the artificial ageing process.

#### 2.1.2. Artificial Ageing of Pristine Microplastics

Artificial ageing of pristine MPs was simulated by photo and thermo degradation. Particles were transferred into Petri dishes and placed in a climate room equipped with UVA lamps, set to specific temperatures, irradiance, and humidity (Table 2). The photodegradation process was induced for a total of 20 days. Afterwards, samples were transferred into an air-circulated oven and exposed at a temperature of 45°C in a dry condition for 25 days. Spectroscopic analyses (ATR-FTIR) determined the functional group change in polymers.

#### 2.1.3. Environmental Weathered Microplastics Collection

River surface microplastic samples were collected from the Ofanto river, the most important river in the Apulia Region (South Italy), during Spring of 2018.

Three surface plankton nets (2.5 × 0.55 m) of 333-μm mesh size fixed in the middle of the river were used to filter water. Material captured by trawl was separated through stainless steel sieves. Material retained on the 0.3 mm sieve was placed in a 40 °C drying oven for 24 h until sample dryness. The biggest plastic particles (5000–2000 µm) were picked manually using forceps and categorized according to colour (transparent, black, blue, and coloured) and morphology as fragments, pellets, and lines.

### 2.2. FTIR Acquisitions

FTIR spectra were acquired using a Nicolet Summit FTIR (ThermoFisher Scientific) equipped with an Everest ATR with a diamond Crystal plate and a DTGS KBr detector (wavenumber range of 4000–500 cm^−1^, 32 scans per spectrum, spectral resolution of 4 cm^−1^). The background was measured with the same settings against air. Ten different PE pellets, fragments, and lines extracted from the Ofanto river were acquired one by one. Comparisons among virgin MPs pellets purchased from the local industry and weathered MPs collected in the river were also made to identify and characterize any changes in the spectra.

### 2.3. Degradation Indexes Calculation

The degree of ageing was evaluated using three different indexes known to be related to changes in MPs: Carbonyl Index (CI), Hydroxyl Index (HI), and Carbon-Oxygen Index (COI).

Carbonyl Index was calculated as the ratio of the absorbance of carbonyl groups at 1770–1700, 1715–1735, 1635–1650 cm^−1^, and at 1495–1423, 1460, and 1452 cm^−1^ for reference peak of PE (adapted from [2]), PP [33] and PS [34], respectively.

In the same way, Hydroxyl Index was calculated as the absorbance ratio of hydroxyl groups at 3353–3021, 3300–3400 cm^−1^, and at 1504–1467 and 986–952 cm^−1^ for reference peak of PE and PP, respectively (adapted from [2]). Finally, Carbon-Oxygen Index was calculated as the ratio of the absorbance of carbon-oxygen groups at 924–1197 and 1000–1200 and the value of reference peaks at 2987–2866 and 2885–2940 for PE (adapted from [2]) and PP [34], respectively. The peak area tool in the OMNIC 9.2.86 software was used to obtain the areas used to calculate the indexes. Following the indexes calculation is reported:

➢ CI_PE_ = Abs (1770–1700)/(1495–1423)
➢ CI_PP_ = Abs (1715–1735)/(1460)
➢ CI_PS_ = Abs (1635–1650)/(1452)
➢ HI_PE_ = Abs (3353–3021)/(1504–1467)
➢ HI_PP_ = Abs (3300–3400)/(986–952)
➢ COI_PE_ = Abs (924–1197)/(2987–2866)
➢ COI_PP_ = Abs (1000–1200)/(2885–2940)

## 3. Results

### 3.1. Artificial Aged MPs

The artificial ageing process of pristine MPs induced surface alterations (e.g., surface cracks, roughness) and changes in the FTIR spectra acquired before and after the artificial weathering process of particles.

**Figure 1:**
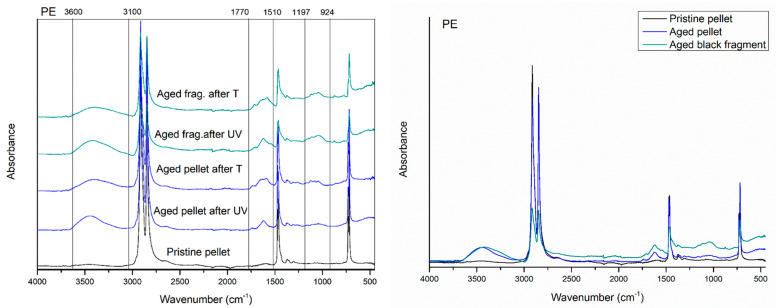
Comparison between the spectrum of the PE pristine pellet (black line), aged pellet (blue line), and aged black fragment (green line), after 20 days of photo-oxidation and 25 of thermos ageing in controlled conditions.

**Figure 2:**
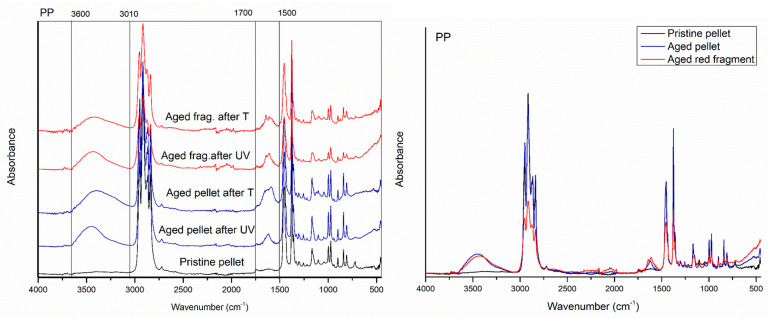
Comparison between the spectrum of the PP pristine pellet (black line), aged pellet (blue line), and aged red fragment (red line), after 20 days of photo-oxidation and 25 of thermos ageing in controlled conditions.

**Figure 3:**
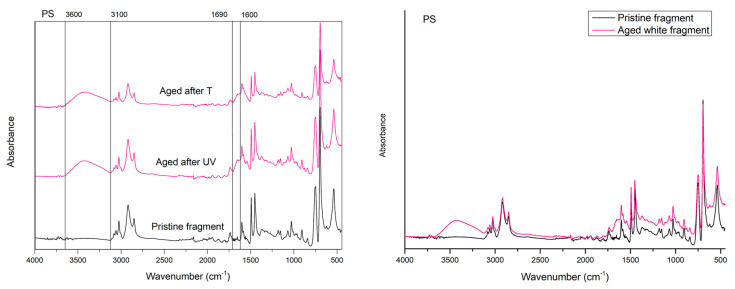
Comparison between the spectrum of the PS pristine fragment (black line) and aged white fragment (red line), after 20 days of photo-oxidation and 25 of thermos ageing in controlled conditions.

The first evident difference in the IR spectra of artificial weathered PE, PP, and PS compared to the raw materials is the alteration of the region relative to the OH stretching (3000 to 3600 cm^−1^), with new absorption peaks appearing in all the spectra polymers after 20 days of UVA exposition in the climate room (Figure 1, Figure 2 and Figure 3).

In the spectra related to the PE fragments and pellets, new weathering bands from 1510 to 1770 cm^−1^ of absorbance, with centred peaks at about 1614 cm^−1^ (corresponding to C=O stretching), are also evident.

A significant increase in the absorbance signal is also notable in the region ranging from 900 to 1200 cm^−1^ showing a higher intensity for the PE fragments than the pellets (Figure 1).

After twenty days of photo-exposition of particles in the climate room, the PP showed new absorption peaks in the carbonyl and double bond region centred at about 1643 cm^−1^ for the fragment and 1578 and 1622 cm^−1^ for the pellet (Figure 2).

Finally, new peaks at 1653 cm^−1^ also appear in the IR spectrum of UV-aged PS fragments (Figure 3).

After 25 days of incubation of particles at 45 °C, PE, PP, and PS materials showed no significant changes in the spectroscopic profile compared to the UVA exposition (Figure 1, Figure 2 and Figure 3).

The information obtained from the IR spectra allowed the calculation of the carbonyl (CI), hydroxyl (HI), and carbon-oxygen (COI) indexes representative of the degree of degradation of particles.

Table 3 and Table 4 show the values related to the different degradation indexes calculated for each particle after 15 and 20 days of exposure to UVA rays (photo-ageing) and following incubation at 45 °C for 25 days (thermo-ageing).

The PE particles showed values of CI increased already after 20 days of UVA exposure, as well as the HI and COI indexes. A further enhancement of values is possible following the incubation of PE particles at 45 °C, where an increase in especially the HI from 7.00 − 7.89 ± 0.23 − 3.79 to 8.15 − 8.21 ± 2.11 − 1.95 is notable.

In the PP particles, the indexes’ remarkable values are notable, particularly for the HI already after 15 days of photo-exposition and for the PS fragments. The CI increases after 20 days of incubation in the climate room, reaching a mean value of 0.71 ± 0.51.

Otherwise, the following incubation of particles at 45 °C further slightly increased the values of indexes; the increase is particularly notable from the value of the HI index of PE particles (both pellets and fragments) and PP fragments (Table 4) which show values of 8.15 ± 2.11, 8.21 ± 1.95 and 25.36 ± 9.01, respectively.

### 3.2. Environmental MPs

In the present work, changes in the surface composition and the level of degradation of particles of various colours and shapes, subjected to different weathering conditions (natural and artificial), were compared to pristine pre-production standard materials.

**Figure 4:**
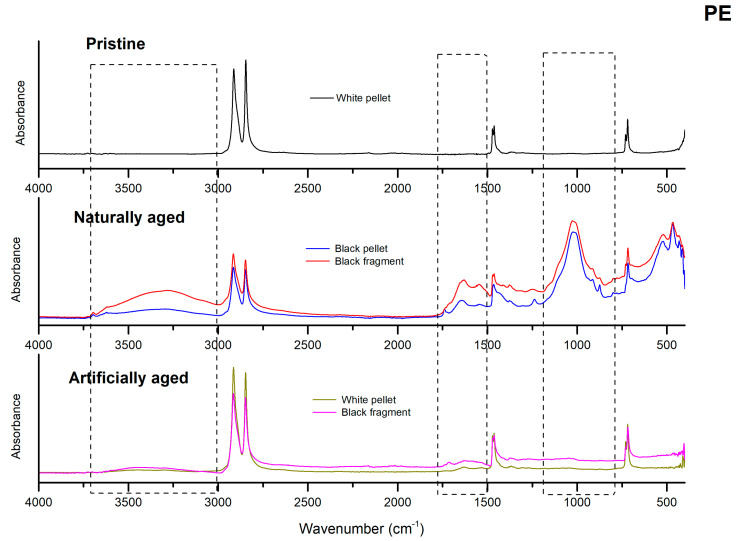
Comparison among the spectra of the PE pristine, artificial aged and naturally weathered particles.

Unfortunately, it was impossible to compare each particle directly for polymer, morphology, and level of ageing due to the scarcity of natural particles detected. Indeed, the vast majority of environmental particles detected in the Ofanto river were composed of PE [4]. Therefore, a direct comparison of natural and artificial weathered and pristine particles has been possible just for PE pellets and fragments (Figure 4, Table 5).

**Figure 5:**
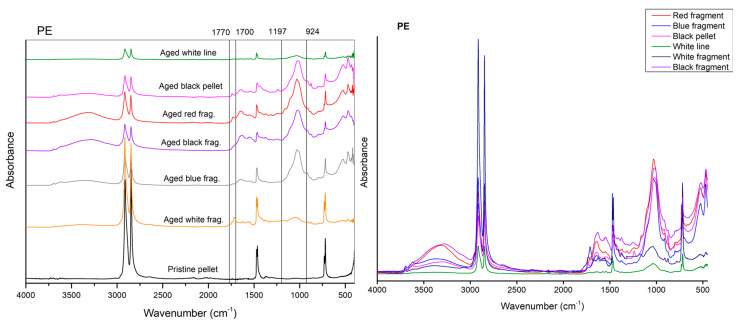
Comparison among the spectra of the PE natural weathered particles.

The results obtained on environmental aged particles showed that the regions reflecting ageing-related changes (hydroxyl groups, broad bands from 3100 to 3700 cm^−1^, alkenes or carbon double bonds, 1600 and 1680 cm^−1^, and carbonyl groups, 1690 and 1810 cm^−1^) appeared significantly modified in the PE environmental particles (Figure 5) compared to the pristine materials and the artificially weathered PE MPs [35].

Moreover, new peaks are present almost in the fingerprint region (1500–500 cm^−1^). Differences in CI, HI, and COI were evidenced among the different morphological MP shapes.

On the one hand, the CI calculated for the PE pellet samples showed values ranging from 0.05 to 0.26 with a mean value of 0.17 ± 0.10. The majority of samples (57%) presented a CI with values between 0.16 and 0.30, defined as medium by [33]. On the other hand, fragments presented slicer modifications in the carbonyl region with CI values lower than pellets (0.05 ± 0.05). Conversely, fragments showed greater values of HI (5.90 ± 2.57) and COI (1.04 ± 0.48) than pellets, as well as lines, which presented the maximum value of HI (11.51).

Comparing the degradation indexes results of PE environmental and artificial aged pellets (Table 5), the values are considerably higher for the formers than for the latter. This is particularly evident for the COI, which more than tripled for the natural pellets (3.11 ± 1.50) and fragments (3.21 ± 1.44) collected from the Ofanto River. The high value of the HI (20.55 ± 17.43) showed for the natural black fragments is also noteworthy.

## 4. Discussion

This study compares the surface degradation process of pristine particles, artificially weathered materials of microscopic size and naturally environmentally aged MPs through spectroscopy investigations.

**Figure 6:**
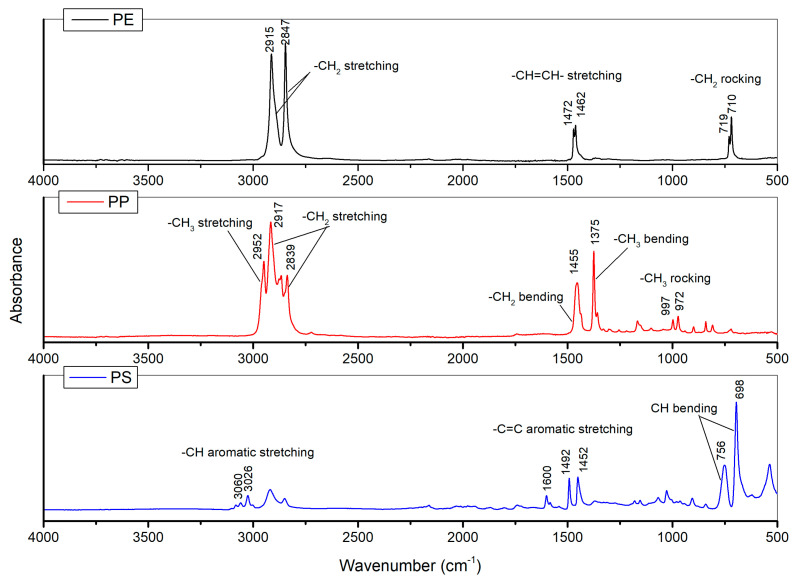
Spectra of pristine PE, PP, and PS particles with characteristics peaks marked for each polymer.

The pristine reference materials had characteristic absorption bands (Figure 6). In particular, for PE at 710 and 719 cm^−1^ (–CH_2_ rocking deformation), 2847 and 2915 cm^−1^ (–CH_2_ symmetric and asymmetric stretching), 1462 and 1472 cm^−1^ (–CH=CH– stretching) [36]. The PP showed reference peaks displayed at 972, 997, and 1165 cm^−1^ (–CH_3_ oscillating vibrations), 1375 cm ^−1^ (–CH_3_ symmetric bending vibrations), 2952 cm ^−1^ (–CH_3_ asymmetric stretching vibrations), 1455, 2838, and 2917 cm^−1^ (–CH_2_ symmetrical bending, stretching, and asymmetrical stretching, respectively) [37].

Finally, the PS was characterized by absorption bands at 3060, 3026 (–CH aromatic stretching vibrations), 1600, 1492, and 1452 (–C=C aromatic stretching), indicating the existence of benzene rings. Otherwise, peaks at 756 and 698 due to C–H out-of-plane bending vibration indicate that only one substituent in the benzene ring is present [38].

In agreement with previous works, the degradation of the pristine materials under different conditions (photo and thermal oxidation) and the environmentally weathered particles resulted in the formation of new functional groups such as carbonyls (1700–1760 cm^−1^), hydroxyl (3300–3400 cm^−1^), vinyl (1600–1680 cm^−1^), peroxides (1100–1300 cm^−1^), and unsaturated groups (880–920 cm^−1^) [39,40,41].

The calculation of degradation indexes lets us quantify the presence of hydroxyl (HO), carbonyl (C–O), and carbon-oxygen bonds (C–O).

Indeed, the CI helps evaluate the degradation of MPs by UV light and their surface oxidation level; it increases with enhancing residence time in the environment [42]. All ketone, carboxylic acid, and ester functional groups formed upon solar irradiation contribute to an increase in the carbonyl signals in the 1650–1850 cm^−1^ region [43].

HI is attributed to the bond vibrations of hydroxyl, carboxyl, or phenol groups. In contrast, COI is frequently attributed to the vibrations of C–O bonds found in carbohydrates and alkanes, secondary alcohols, and ketones [2,34].

The indexes calculated for polymers degraded under the artificial photo and thermo-ageing conditions displayed a general tendency of the indexes to increase with the time in contact with irradiation time. Indeed, after 20 days of exposition to UVA, particular enhancements of CI of PS fragment and PE pellet, HI of PE and PS fragments and PE pellet, and COI of PS fragment were observed. When UV radiation meets with plastic, the polymer’s photo-oxidation occurs, resulting in the scission of C-H bonds and free radicals production [44].

Otherwise, the following incubation of the same particles for 25 days at a constant temperature of 45 °C does not seem to have further altered the chemical composition of the particles; with just some increase in the HI index (e.g., for PP pellets) observed. Indeed, as also observed by other authors [45], the degradation degree of UV ageing is confirmed to be greater than that of thermal weathering. Moreover, the thermal degradation of plastic in the environment is unlikely to happen due to the high temperature required to start thermos-oxidative reactions. Instead, a slow thermal degradation can occur in synergy with photodegradation, especially in environments directly exposed to sunlight (e.g., beaches, and soils) [8].

Considering the comparison between PE artificial and naturally weathered particles of different colours and morphologies, we can observe the highest value of CI calculated for the white fragments spectra (0.34). A CI value above 0.30 is considered high by [33]. This result suggests a possible greater residence time of these particles in the environment with respect to the other coloured pellets and fragments detected.

A similar study [42] conducted in the marine environment of Cartagena (Spain), showed comparable values of CI calculated in MPs collected in coastal and marine sediments. The former were significantly higher than the latter, with values ranging from 0.28 to 1.57 for the beached particles and from 0.21 to 0.51 for the submerged particles (collected from marine sediments).

Moreover, the CI values were higher in the coastal sediments close to the high-tide line due to the exposition of the wave action over the solar and microorganisms exposition. Indeed, as also mentioned by other authors [46], plastic degradation is more rapid on land than in water, where exposure to solar radiation and mechanical erosion is minimal.

Another study [43] also evaluated the carbonyl index of meso and microplastics collected in the North Atlantic sub-tropical gyre. The authors found a CI value for PE particles not exceeding 0.7. Such carbonyl values, observed in our study and previous ones, can be explained by the fact that photo-oxidation is generally limited to the surface layer of polymers (100 µm) due to the high polymer crystallinity that, leading light to high scattering and reflection, reduces its distance of penetration. Therefore, the hydroxyl radicals produced are confined to the external polymer surface; with the increasing UV-exposition, the upper layer of plastic becomes so fragile that it erodes and leaves a non-oxidized layer exposed and ready to be oxidized again. This could explain why the carbonyl indices do not exceed certain values [43,47].

Even the colour of these particles confirms what has been detected; in fact, the white colour of these particles is an indication of a loss of colour, the same as which led to a general yellowing probably caused by fragmentation of macro and mesoplastics present in the environment, which occurred over a considerable time. Otherwise, coloured environmental weathered fragments showed considerably lower CI values than the white ones, suggesting younger particles. However, a medium value of CI [33] equal to 0.26 has also been calculated for black environmental-aged pellets. Furthermore, considering the artificially aged particles, black fragments showed a higher CI than white pellets. 

We can explain these deviations of CI based on differences in crystallinity or density of the PE particles. It is known that the more amorphous the polymer material, the easier the diffusion of oxygen and the higher the CI.

Indeed, several types of polyethylene polymer are synthetized from chemists (Low-density polyethylene LDPE, high-density polyethylene HDPE, linear low-density polyethylene LLDPE) exhibiting a random orientation of side chains for LDPE, linear chain for HDPE and short side chains for LLDPE. The difference in the PE chains’ linearity translates into different crystallizing properties [17,41,48].

**Figure 7:**
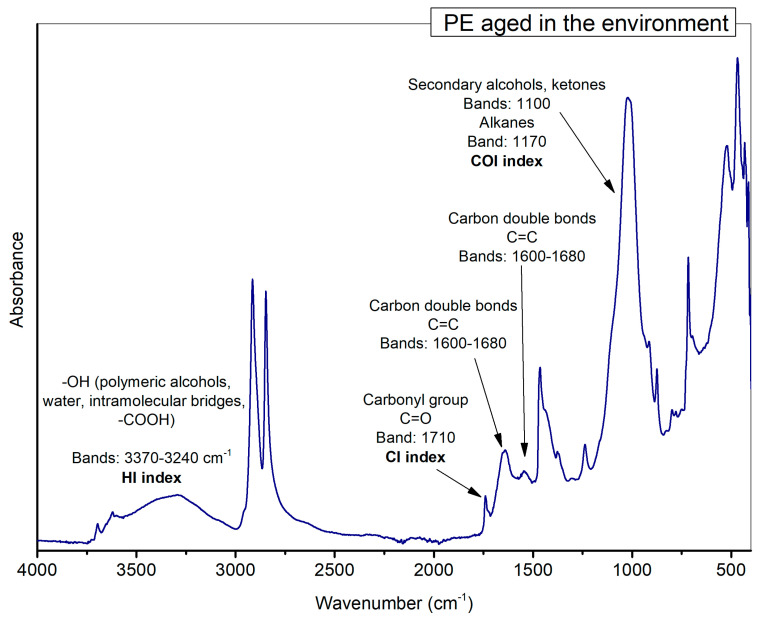
Example of a spectrum of naturally weathered PE with the appearance of new functional groups due to degradation process.

Large absorption peaks appeared in the region of OH stretching (centred at about 3400 cm^−1^) of artificially aged polymers and all PE samples collected from the Ofanto river (Figure 4 and Figure 7). This absorption band, highlighted at high signals in many spectra but more intense for the environmental samples, could be attributed to the hydrolysis reaction that occurred in the polymer chains with an increase in hydroxyl groups due to the effect of the permanence time in the environment [49]. Moreover, as suggested by [6,50], the consequent formation of surface cracks and fractures, increasing with the environmental exposition, could allow a more accessible and deeper infiltration of water and oxygen from the surrounding ambient, leading, in time, to an increased effect of ageing. The hydroxyl content of environmentally weathered PE particles showed a certain degree of variability among the particles of different colours and shapes. The diversity in hydroxyl band intensity is possibly due to the non-homogenous weathering that occurs on the particles related to their different source of origin; otherwise, it could be a result of exfoliation and flaking of the plastic surface. Previous research also detected changes in this absorption area in particles aged under artificial conditions and observed more marked peaks in samples weathered in water than compared to those in the air [51].

Other authors also evaluated filmy microplastics extracted from paddy soil and assessed their ageing properties by calculating carbonyl and hydroxyl indexes. Carbonyl and hydroxyl indexes increased, especially in soils continuously mulched for ten years, showing values consistent with ours [45].

Intense peaks appeared in our samples, between 1000–1200 cm^−1^ (centred at about 1029 cm^−1^), indicating the spectral range of carbon-oxygen bonds, particularly relevant just in environmental weathered particles (Figure 7). The region at 1000–1200 cm^− 1^ is attributed to the stretching vibration absorptions of the C-O (around 1160 cm^−1^), indicating different C-O-containing degradation products but may also indicate phosphate ions, bonds of the glycosidic linkages [52], or aliphatic phosphates. It is interesting to observe the presence of similar peaks (from 1162 to 1035 cm^−1^) in previous work [51] that, comparing the ageing of particles suspended in different matrices (deionized water, artificial seawater, natural seawater, air), detected new unique products in the particles weathered in the natural seawater.

Finally, the region 700–400 cm^−1^, usually called the fingerprint region, appeared considerably different compared to artificially aged and pristine particles; this region is indeed unique for various compounds [53].

These peaks can be attributed to fouling by sand, debris, and organic material (biofilm) absorbed on the polymer’s surface [49] as well as to the presence of additives and plasticizers [4].

Detecting characteristics spectra acquired from environmental particles indicates that artificial weathered polymers do not account for all the observed functional groups and possibly microplastic pollution effects. These differences represent the heterogeneity of the surrounding environment in terms of nutrients, organic matter, conductibility, and microbial environment, indicating the necessity of more studies representing environmental conditions to fully understand microplastics’ ageing processes.

Given the high intensity of fingerprint signals, it is assumed that a considerable amount of material has adhered to the particles sampled from the environment. Indeed, freshwater microplastics most likely originate from terrestrial environments where they are subjected to the mechanical actions of the soil for a long time, resulting in the adhesion of foreign substances to their surface [45].

The major microplastic drivers identified in the terrestrial environment are irrigation water, urban and agricultural runoff, fertilizers, and soil amendments, including sewage sludge, which have to be considered in the counter-measuring policies and institutionalization process [54,55].

Necessary policies and governance measures must support the creation of appropriate barriers to MPs entering humans through food chains.

Indeed, present plastic waste management regulations mainly focus on waste disposal on land. In contrast, the policies regarding MPs have primarily focused on preventing just marine aquatic MP pollution [56].

## 5. Conclusions

The analysis of the spectroscopic investigations carried out by ATR-FTIR, allowed us to assess the level of ageing of particles weathered under controlled conditions of light and temperature and of microplastics collected in a natural freshwater environment such as the Ofanto river.

The spectra analysis of particles aged under controlled conditions let us conclude that UVA radiation (315–400 nm) exposition was the most critical condition influencing and starting the degradation processes of PE, PP, and PS polymers. The calculation of degradation indexes indicated suggested that PE pellets resulted in the most resistant microplastic category to photo and thermo ageing. Otherwise, PS fragments showed the most altered spectra.

By calculating degradation indexes and observing new peaks detected in both artificial and natural aged particles, we can likely conclude a permanence time in the environment of freshwater particles above the total ageing period considered in the laboratory for the weathering (45 days).

Real freshwater microplastic samples showed further alteration of all spectra with the appearance of new unique absorption peaks respected to artificial weathered particles demonstrating that several variables interact together in the weathering process of polymers.

In light of our results, conducting field studies is paramount to thoroughly implementing and revising plastic policies, considering all the environmental variables influencing weathering plastic processes. It is, in fact, known that aged plastic has a greater susceptibility to pollutants adsorption and microbial adhesion. Indeed, one of the primary concerns related to microplastics stems from their capacity to act as carriers of dangerous substances. (e.g., persistent organic pollutants, antibiotic resistance genes, heavy metals, alien species, etc.).

Compared to previous studies, based on experiments that accelerated plastic particle ageing in the laboratory or simulated environmental conditions, our study is the first experiment representing a realistic assessment of the degradation process that occurs on freshwater microplastics.

## Figures and Tables

**Table 1:** Pristine MPs were selected for ageing under controlled conditions.

Polymer	Shape	Colour	Size	Source
Polyethylene	Fragment	Black	5–1 mm	Mulch sheet
	Pellet	White-grey	5 mm	Plastic industry
Polypropylene	Fragment	Coloured	5–1 mm	Plastic cup
	Pellet	White-grey	5 mm	Plastic industry
Polystyrene	Fragment	White	5–1 mm	Box

**Table 2:** Setting Parameters for the artificial photo-ageing process of MPs in the climate room.

Radiation	Wavelength	Temperature	Irradiance	Humidity
UVA	340 nm	22 °C	12 h	60%

**Table 3:** Comparison of CI, HI, and COI calculated for each polymer. Values are expressed as an average of two replicates ± standard deviation (*Ds*) after 15 and 20 days of exposure to UVA rays in a climate room.

	Polymers	CI ± *Ds*		HI ± *Ds*		COI ± *Ds*	
		t_15_	t_20_	t_15_	t_20_	t_15_	t_20_
Photo-Ageing	PE Pellet	0.08 ± 0.10	0.15 ± 0.02	1.61 ± 1.25	7.00 ± 0.23	0.04 ± 0.01	0.10 ± 0.02
	PE Fragment	0.13 ±0.04	0.12 ±0.05	0.22 ± 6.63	7.89 ± 3.79	0.24 ± 0.22	0.26 ± 0.26
	PP Pellet	0.05 0.00	0.05 0.02	18.85 ±21.41	15.17±11.94	0.14 ± 0.05	0.13 ± 0.04
	PP Fragment	0.02 ± 0.02	0.04 ± 0.00	12.43 ±10.55	35.72 ± 4.10	0.14 ± 0.01	0.29 ± 0.16
	PS Fragment	0.24 ± 0.24	0.71 ± 0.51	-	-	-	-

**Table 4:** Comparison of CI, HI, and COI calculated for each polymer. Values are expressed as an average of two replicates ± standard deviation (*Ds*) after 25 days of incubation at 45 °C in a dry condition.

	Polymers	CI ± *Ds*	HI ± *Ds*	COI ± *Ds*
		t_25_	t_25_	t_25_
Thermo-Ageing	PE Pellet	0.11 ± 0.06	8.15 ± 2.11	0.06 ± 0.01
	PE Fragment	0.21 ± 0.04	8.21 ± 1.95	0.20 ± 0.07
	PP Pellet	0.11 ± 0.00	25.36 ±9.01	0.26 ± 0.07
	PP Fragment	0.04 ± 0.03	19.02 ± 10.32	0.16 ± 0.01
	PS Fragment	0.74 ± 0.43	-	-

**Table 5:** Comparison of CI, HI, and COI calculated for freshwater PE particles collected from the Ofanto river and artificial aged PE pellets and fragments.

Polymer	Morphology	Indexes					
		CI		HI		COI	
		Artificial Ageing	Environmental Ageing	Artificial Ageing	Environmental Ageing	Artificial Ageing	Artificial Ageing
Polyethylene	Black pellets	0.11 ± 0.06	0.26 ± 0.05	8.15 ± 2.11	8.51 ± 2.18	0.06 ± 0.01	3.11 ± 1.50
	Black fragments	0.21 ± 0.04	0.19 ± 0.16	8.21 ± 1.95	20.55 ± 17.43	0.20 ± 0.07	3.21 ± 1.44
	White fragments	-	0.34 ± 0.37	-	5.37 ± 3.76	-	2.90 ± 1.88
	White line	-	0.07 ± 0.03	-	1.34 ± 0.93	-	0.91 ± 0.21
	Blue fragment	-	0.05 ± 0.02	-	3.54 ± 1.76	-	1.88 ± 0.84
	Red fragment	-	0.18 ± 0.13	-	17.63 ± 12.92	-	3 ± 1.72

## Data Availability

Not applicable.
